# Biological Insights into Therapeutic Protein Modifications throughout Trafficking and Their Biopharmaceutical Applications

**DOI:** 10.1155/2013/273086

**Published:** 2013-04-18

**Authors:** Xiaotian Zhong, Jill F. Wright

**Affiliations:** Pfizer BioTherapeutics Research & Development, 87 Cambridge Park Drive, Cambridge, MA 02140, USA

## Abstract

Over the lifespan of therapeutic proteins, from the point of biosynthesis to the complete clearance from tested subjects, they undergo various biological modifications. Therapeutic influences and molecular mechanisms of these modifications have been well appreciated for some while remained less understood for many. This paper has classified these modifications into multiple categories, according to their processing locations and enzymatic involvement during the trafficking events. It also focuses on the underlying mechanisms and structural-functional relationship between modifications and therapeutic properties. In addition, recent advances in protein engineering, cell line engineering, and process engineering, by exploring these complex cellular processes, are discussed and summarized, for improving functional characteristics and attributes of protein-based biopharmaceutical products.

## 1. Introduction

Nature has evolved complex biological processes that enable mammalian cells to conduct sophisticated physiological activities for living and adaptation. Primary structure of a protein obtained from genome projects serves as a nice starting point for the understanding of biological complexity but not sufficient to explain various functions and regulations. Majority of proteins from eukaryotic cells are subjected to certain kind of covalent modifications either during or after their ribosomal synthesis. An increasing appreciation of these cellular homeostatic modifications, which have been shown to initiate various biological functions and regulation mechanisms, should contribute to unfolding detailed knowledge about biological networks and living systems. 

One of the earliest pieces of evidences for the existence of posttranslational modifications can be traced back to the finding of protein phosphorylation published in 1883 [1, 2], in which protein casein was found containing stoichiometric amount of phosphate. One of the earliest modified proteins to be studied, the first glycoprotein, is likely the glycogen of liver which the French physiologist Claude Bernard famously identified as “glycogenous matter” in 1855 [3]. During the biosynthesis of all proteins, the building blocks are 19 standard amino acids (plus selenocysteine for a few selenoproteins) and one imino acid. Nonetheless upon hydrolysis, close to 200 different amino acids have been identified, indicating a significant posttranslational modification of the originally encoded sequences [4]. Over the years, more than 200,000 reported modification events have been unveiled by a PubMed analysis [5]. Several hundred modifications have been characterized to date [4, 6, 7]. Some of these modifications are spontaneous reactions, while most of them involve specific enzymes and pathways. Certain structural determinants within the primary sequences of polypeptides are recognized by cellular machineries, and are carefully regulated by time and space. Efficiency of these modifications varies according to cell types, availability of substrates and cofactors, and biological conditions.

Most therapeutic proteins approved or in development bear at least one or more of posttranslational modifications [8–10]. Majority of these proteins are originally synthesized in endoplasmic-reticulum-(ER-) bound ribosomes, translocated across ER membranes through translocon, and transported through the secretory pathway into extracellular space. A subset of modifications accompany along with this biological process, and additional modifications occur during *in vitro* manipulations, that is, purification, formulation, storage, and injection into test subjects. These modifications and their underlying molecular mechanisms form the main focus of this review. Other modifications that are characteristics of intracellular proteins, such as acetylation, ADP ribosylation, sumoylation, and ubiquitination, can be found in several recent reviews [5–7, 11–14] and therefore not considered in this paper. 

**Figure 1:**
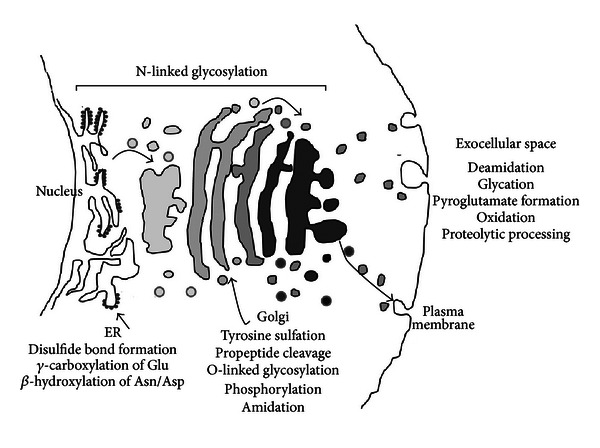
Classification of therapeutic protein modifications along trafficking pathways.

It is obvious that posttranslational modifications affect structural and functional aspects of therapeutic proteins. The effects can be detrimental, that is, heterogeneity [15] and immunogenicity [16], even though the modification may originally be required for functional activity of the polypeptide. A better understanding of the relationship between the primary sequences of therapeutic proteins and cellular machineries can allow developers to avoid unwanted side effects of these modifications. Such knowledge can also help improve protein efficacy and quality through protein engineering, cell line engineering, and process engineering. This review classifies these modifications into four major categories, three of which are based on the locations where these modifications take place within the trafficking events: ER, Golgi, and Exocellular space (Figure 1). N-linked glycosylation modification involves both ER and Golgi compartments, therefore being categorized as a separated group. In each of these categories, molecular mechanism and specific pathways for each modification are described (Table 1). The purpose of the review is restricted to modifications that may be found on therapeutic proteins secreted from animal cells. Therapeutic protein examples bearing the corresponding modification are discussed, and their effects on physiological and biophysical properties are analyzed. In the last part of the paper, recent progresses on protein engineering, cell line engineering, and process engineering, by utilizing posttranslational modification pathways, are considered and summarized (Table 2).

## 2. ER Modifications

During biogenesis in eukaryotic cells, precursor forms of therapeutic proteins are translated in ribosomes, which are targeted to rough ER through a hydrophobic signal sequence bound by a signal recognition particle ribonuclear protein complex [17]. Depending on the overall amino acid content of the protein, the translocation across ER membranes can occur co- or posttranslationally, both of which require protein translocon, the Sec61 complex [18]. In ER lumen, the protein has its signal peptide removed via membrane-bound signal peptidase, gains proper folding through ER luminal folding machinery, is subjected to ER quality control process, and transports to next secretory compartment. Several modifications take place for therapeutic proteins when they transit through this compartment. These modifications include disulfide bond formation, gamma carboxylation, beta hydroxylation, and N-linked glycosylation which is discussed in the latter section.

### 2.1. Disulfide Bond Formation

Disulfide bond formation is one of the most common modifications found in extracellular proteins. Therapeutic proteins, such as antibodies, coagulation factors, and insulin, contain both interchain and intrachain disulfide bonds. Disulfide bond formation helps stabilize tertiary structures and is important for assembly and maintenance of protein structural integrity. Without disulfide bonds, native conformations for these proteins are not obtained. For multi-unit proteins such as antibody, interchain disulfide bonds help connect multiple protein subunits together during polypeptide assembly.

Disulfide bond formation takes place in ER lumen where it is much more oxidizing than cytosol as measured by reduced-oxidized glutathione couple [19]. A highly specific and targeted redox protein network maintains this redox homeostasis and ensures proper disulfide bond formation [20, 21]. To attain a given conformation, native and nonnative disulfides are transiently formed and reduced. A precise equilibrium between oxidation and reduction reactions in ER is required to make certain that these covalent links remain dynamic until folding is completed. Either a reducing ER, preventing disulfide formation, or an overoxidizing ER, stabilizing nonnative bonds, can trigger ER stress responses [22]. 

Molecular pathways for disulfide bond formation have been discovered in eukaryotes and prokaryotes [21, 23]. Both systems share a common design, including the transfer of thiols between membrane-associated oxidoreductases to soluble oxidoreductases and direct introduction of disulfide bonds into substrate proteins. In mammalian cells, membrane-associated flavoprotein Ero1 utilizes the oxidizing power of molecular oxygen, coupled with its flavin cofactor, to generate disulfide bonds within itself, which are transferred to protein disulfide isomerase (PDI). Then PDI transfers its disulfide bonds directly to secretory proteins in ER. Both human and yeast cells contain multiple PDI-like proteins. These PDI homologs may contribute to discrete protein maturation pathways in different cell types [21]. Besides the Ero1-dependent pathway, alternative oxidative folding pathways, such as the quiescin sulphydryl oxidase/Erv superfamily and vitamin K epoxide reductase, also contribute to the cellular disulfide bond formation process [24]. 

Incomplete formation of disulfide bonds can cause issues like heterogeneity and disulfide bond scrambling for therapeutic proteins [15]. It has been reported that a significant fraction of free sulfhydryl and reactive cysteine residues is found in recombinant antibodies of IgG1, IgG2, and IgG4 [25]. Interchain disulfide bonds between heavy chains or between heavy chain and light chain are susceptible to reduction [26], while intrachain disulfide bonds within each IgG domain can only form free sulfhydryl groups due to incomplete bond formation during biosynthesis. This formation of free sulfhydryl group can increase molecular mass by 2 Da and result in size heterogeneity. Presence of incomplete disulfide bonding can induce disulfide bond scrambling and cause structural disturbances. Human IgG2 antibodies contain three types of disulfide isoforms classified by the disulfide links between Fab arms and heavy chain hinge region, contributing to IgG2 heterogeneity [27]. Human IgG4 is also known to have nonclassic disulfide bond heterogeneous structure, with an equilibrium between forming two inter heavy chain disulfide bonds and forming two intrachain disulfide bonds of half-molecules [26]. Trisulfides, rare variants of disulfides that contain an extra sulfur atom, are reported to be detected in IgG antibodies [26, 28]. The formation mechanism is unclear, possibly due to *in vitro* protein manipulation as a result of the reaction of an intact disulfide bond with dissolved hydrogen sulfide [26, 28]. 

### 2.2. Gamma Carboxylation of Glutamate Residues

*γ*-carboxylation is a type of modification with a conversion of target glutamate (Glu) residues to *γ*-carboxyglutamate (Gla), first discovered in 1974 in Prothrombin [29, 30]. This process is undertaken by a vitamin K dependent carboxylase (GGCX), an ER bound integral membrane protein with at least five transmembrane domains [31, 32]. In the presence of CO_2_, O_2_, and vitamin K hydroquinone, GGCX converts glutamate residues to Gla residues, yielding vitamin K epoxide and H_2_O. The vitamin K epoxide is subsequently reduced to regenerate vitamin K hydroquinone by vitamin K epoxide reductase (VKOR) [33].

*γ*-carboxylation is a characteristic of a group of proteins termed vitamin K dependent proteins that play roles in blood coagulation and bone metabolism [34]. They contain this so-called Gla domain that possesses 9–12 Gla residues in a roughly 45 amino acids peptide sequence. Gla residues provide the calcium binding ability to the Gla-domain-containing proteins. This activity induces conformation changes for interacting with membrane phospholipids. Undercarboxylation of these proteins can severely impair their biological activities. Gla residue is not reused for protein synthesis but is excreted in urine for mammals [34]. Carboxylation of Gla domain is not dependent on a specific consensus sequence, but mediated by an immediately adjacent propeptide region. The propeptide contributes to docking protein substrate to GGCX and may also serve as an allosteric effector in* trans * [34].

Currently three therapeutic proteins are *γ*-carboxylated: factor IX, VIIa, and activated protein C. Another *γ*-carboxylated protein thrombin is used as a topical treatment for breeding control [35]. The recombinant factor IX contains 12 glutamic acids that can be carboxylated. High-level expression of factor IX in Chinese Hamster Ovary (CHO) cells decreases the carboxylation rate and therefore results in a much lower specific activity when compared to the natural human plasma-derived protein [36]. Factor X was found only 30% fully carboxylated when expressed in human embryonic kidney cells [37], likely due to the saturation of GGCX enzyme. Interestingly, replacing factor X propeptide with that of prothrombin, a 43-fold lower affinity substrate for GGCX, results in 90–95% of factor X being fully carboxylated. Factor VIIa contains 10 carboxylation sites that are important for its biological activities [38], and the recombinant version with 9 out of 10 sites carboxylated retains full activity. Activated protein C that is used to treat severe sepsis has 9 carboxylation sites, and full carboxylation of all sites is required for its biological activity [39].

### 2.3. Beta Hydroxylation of Aspartate and Asparagine

*β*-hydroxylation was first discovered in early 1980s from bovine protein C [40]. It is undertaken by hydroxylase enzymes that convert target aspartate (Asp) residues to *β*-hydroxyaspartate (Hya) or asparagine (Asn) residues to *β*-hydroxyasparagine (Hyn) [41–43]. This modification has also been shown to bind calcium and to be involved in protein-protein interaction.

*β*-hydroxylation takes place in ER and requires 2-ketoglutarate and Fe^2+^ [41, 43]. This modification is catalyzed by a type II membrane protein *β*-hydroxylase with a C-terminal catalytic domain in ER lumen [42]. The reaction does not require vitamin K or an adjacent propeptide. A consensus *β*-hydroxylation site (Cys-X-Asp/Asn-X-X-X-X-Phe/Tyr-X-Cys-X-Cys) found in EGF domain was proposed [44]; however, proteins like factor VII with such a site is not hydroxylated, suggesting that further requirements are needed [38]. 

*β*-hydroxylation is found in epidermal growth factor (EGF) precursor and in some calcium-binding EGF-like domain receptors, ligands, extracellular matrix proteins, complement proteins, and vitamin K dependent homeostatic proteins [35]. It occurs in therapeutic protein factor IX and protein C. Both plasma-derived factor IX and recombinant factor IX from CHO cells are similarly hydroxylated at Asp64 (~30–40%) [45]. Hydroxylation seems to be nonessential for the clotting activity. On the other hand, hydroxylation of activated protein C at Asp71 is essential in maintaining its biological activity in factor Va and factor VIIIa inactivation [10, 39].

## 3. Golgi Modifications

Golgi complex is well established as a central processing and sorting station for protein trafficking [79] and as an organelle with multiple complex functions [80]. In mammalian cells, Golgi stacks are laterally linked to form a ribbon-like membrane system. When neosynthesized and properly folded soluble therapeutic proteins exit ER, membrane vesicles containing these proteins fuse with a network of vesicular tubular clusters known as ER-Golgi intermediate compartment that sorts proteins to cis-Golgi or carries them back to ER. While the Golgi-arrived proteins transverse Golgi cisternae and sort further at trans-Golgi network to various cellular destinations, they are subjected to a number of modifications.

### 3.1. Tyrosine Sulfation

Sulfation addition to tyrosine of secretory proteins as an O^4^-sulfate ester takes place in trans-Golgi [81]. This modification was first discovered in 1954 in a bovine fibrinogen peptide [82]. Since then hundreds of tyrosine-sulfated proteins have been reported [83–86]. The modification is mediated by two closely related integral membrane protein tyrosylprotein sulfotransferases (TPST1 and TPST2) [87–89], with the catalytic domain orientated in the lumen of trans-Golgi [90, 91]. Orthologues of TPST1 and TPST2 have been found in both vertebrate and invertebrate [84], supporting functional importance of this modification, although no tyrosine sulfation occurs in yeast or prokaryotes.

The reaction catalyzed by TPSTs employs 3′-phosphoadenosine 5′-phosphosulfate (PAPS) as sulfate donor [83], in which the sulfate from 5′-phosphosulfate group of PAPS is transferred to the phenol of a tyrosine residue to form an O-sulfated tyrosine and adenosine 3′,5′-diphosphate [84]. There is no simple consensus sequence for tyrosine sulfation, although certain common features have been identified based on the sequence analysis with known sulfated tyrosines that seem to favor this modification. One of the major characteristics is the presence of acidic amino acids, such as aspartic or glutamic acid, adjacent to tyrosine at N-terminal end [81, 87, 92]. Turn-induced amino acids around tyrosine residue, as well as lack of cysteine and glycosylation sites, favor sulfation of nearby tyrosine residues [81, 92]. 

For therapeutic proteins, tyrosine sulfation is required for factor VIII's full procoagulant activity, including conversion of factor VIII to VIIIa and optimal binding to von Willebrand factor [36]. Tyrosine sulfation at 155 in factor IX influences factor IX's *in vivo* recovery [36]. The functional importance of tyrosine sulfation appears to be modulating biological activity and binding affinities by affecting protein-protein interaction [86]. Many chemokine receptors, G-protein-coupled receptors of complement proteins, phospholipids, and glycoprotein hormone receptors, have been shown to be tyrosine sulfated, which play an important role in their high affinity ligand binding and subsequent receptor activation. C-terminal tyrosine sulfated hirudin displays tenfold higher affinity to thrombin than the unsulfated version [93–95]. Tyrosine sulfation also appears to affect protein stability, as human secreted fizzled-related protein-1 is less stable with the presence of sulfation in its tyrosines 34 and 36 [96]. 

### 3.2. Propeptide Processing

Many bioactive proteins and peptides are synthesized as an inactive precursor which is activated by limited proteolysis. The specific processing of these proproteins and prohormone peptides occurs at relatively conserved sites often with single or pairs of basic amino acids [97, 98]. A special family of proteases, termed preprotein convertases (PCs), process these inactive precursors while they traffick through the secretory pathway. Seven members of this PC family are basic amino acid specific mammalian proteases that are known as PC1/3, PC2, Furin, PC4, PC5/6, PACE4, and PC7 [98]. The eighth member of the family is SKI-1 that cleaves substrates at the motif (R/K)-X (hydrophobic)-X↓ [99]. The latest member of the family is PCSK9 that autocatalytically cleaves its propeptide at VFAQ152↓ [100]. 

All these convertases themselves are synthesized as inactive zymogens. Through an autocleavage event in ER or in immature secretory granules (for PC2 only), an inactive heterodimer of inhibitory propeptide associated with the rest of the protein is formed. After exiting ER, a second autocatalytical event occurs on the prosegment at specific subcellular compartments along the secretory pathway to liberate the active enzyme free from the inhibitory propeptide [98]. This activation mechanism ensures that these PCs are only active at specific intracellular sites. SKI-1, Furin, PACE4, PC5/6, and PC7 are active at the Golgi complex, PC1/3 and PC2 are maximally active in secretory granules, while PCSK9 is the only PC that is secreted as catalytically inactive heterodimer [98]. 

A number of therapeutic proteins or peptides are synthesized as proproteins. Proinsulin is processed by either PC1 or PC2 [97]. Bone morphogenetic protein-2 precursor [101] is specifically processed by PC5/6 [102]. The presegments of both Pro-factor VII [97] and Pro-factor IX [103] are processed by Furin. Failure to remove the propeptide from Pro-factor IX can result in a secretable molecule that is defective in phospholipid interaction and displays reduced *γ*-carboxylation of glutamic acid residues [104]. Factor VIII is processed at arginine 1313 and 1648 of its B-domain which has consensus site for Furin, though the exact PC members responsible for this processing remain unidentified [105]. 

### 3.3. O-Linked Glycosylation

In contrast to N-linked glycosylation which initiates cotranslationally in ER lumen with a transfer of a core glycan chain from dolichol-pyrophosphate, O-linked glycosylation in mammalian cells begins in Golgi apparatus with addition of single monosaccharide from sugar nucleotide precursor [106, 107]. Several types of O-linked glycosylation can be classified. Mucin-type glycans have N-acetylgalactosamine (GalNAc) attached to hydroxyl group of Ser or Thr, which are found on many secreted and membrane-bound glycoproteins. No particular sequence motif has been identified for mucin-type O-linked glycosylation, except with proline favorable and charged amino acids interfering nearby specific sites [106, 107]. Elongation of this GalNAc generates at least eight different core structures. Some other specific types of O-linked glycosylation have also been identified. O-linked fucoses and glucoses are found on a specific consensus sequence in EGF protein domains [108]. One special O-linked glycosylation is galactose (Gal) on hydroxylysines of collagen in the sequence of Gly-Xaa-Hyl-Gly- [109]. Another special kind of O-linked glycans is glycosaminoglycans (GAGs) that are typically found in proteoglycans produced virtually by all mammalian cells [110]. GAGs are linked to serine residues in core protein by xylose. Xylosyltransferase initiates this modification in Golgi at specific sites defined by Ser-Gly flanked with one or two acidic residues. 

Mucin-type of O-linked glycosylation can be often found in therapeutic proteins. The first step for this modification is the transfer of GalNAc to Ser or Thr, catalyzed by a polypeptide-N-acetyl-galactosaminyltransferase (ppGalNAcT). There are at least 21 ppGalNAcT homologs expressed in eukaryotic organisms [111]. ppGalNAcT expression and its Golgi sublocalization vary considerably between cell types and tissues, contributing partly to heterogeneity of O-linked glycans. Further elongation and termination of O-linked glycans after addition of the first GalNAc can be synthesized by various glycosyltransferases. Expansion with galactose, GlcNAc, or GalNAc produces eight different core structures. The glycans can be additionally modified by sialylation, fucosylation, sulphatation, methylation, or acetylation, which lead to a large number of oligosaccharide structures [107, 111].

Like N-glycans, O-linked glycans play a role in maintaining structures of fully folded proteins, modulating aggregation, maintaining protein stability, and conferring protease and heat resistance. O-linked glycan modifications have been found in hinge region and possibly Fc region for a number of antibodies [15]. The hinge region modification can render protease resistant for antibody. Examples include modifications at the hinge regions of IgG2b [112], IgA_1_, and IgD [107, 113]. O-linked modification in human erythropoietin (EPO) is not essential for its activity [114]. O-linked glycans are also found in the activation peptides of factor V and VIII and in the EGF domain of factor IX, VII [36], tissue plasminogen activator [115], and recombinant human urokinase [116]. The functional significance of these glycans remains unclear. One of the adverse effects of O-linked glycans on therapeutic proteins is heterogeneity [15, 107]. Recently O-linked glycans are found inhibiting the binding activity and efficacy of a therapeutic peptide-antibody bispecific fusion by blocking its interaction with cytokine IL17A [53].

### 3.4. Phosphorylation

Intracellular protein phosphorylation has been well documented as a fundamental mechanism regulating many essential cellular processes; yet physiological importance and functional regulation of extracellular phosphorylation on extracellular proteins remain obscure comparatively. Recent proteomic and computational analysis has revealed a large number of extracellular phosphorylated proteins and phosphorylated sites [117]. It also provides evidence that extracellular phosphorylation plays a role in various physiological functions, such as blood coagulation, formation of neuronal networks, immune cell activation, and biomineralization [2, 117, 118]. 

It has been long assumed that the concentration of ATP is not sufficiently high in extracellular environment, even though constitutive release of ATP extracellularly has been observed in many cell types [119–121]. The most recent proof for extracellular phosphorylation events is the identification of two Golgi-localized ectokinases, four-jointed [122], and FAM20C [2, 118]. Both proteins contain an N-terminal signal sequence and a kinase domain. FAM20C has been demonstrated to be the long-sought “Golgi-enriched fraction casein kinase” (GEF-CK) that phosphorylates many extracellular proteins. The consensus site recognized by GEF-CK is S-x-E/pS, where X can be any amino acid and E/pS can be Glu or phosphoserine [2, 118]. Another FAM20 member, FAM20B, has been identified as an ectokinase that phosphorylates xylose within GAG core linker [124]. These findings are consistent with the notion that extracellular phosphorylation mainly takes place in the Golgi compartment, where sufficiently high concentration of luminal ATP provides phosphorylation substrates and is the source of constitutively released ATP into the extracellular space of many cell types [121].

A number of therapeutic products or candidates have been found phosphorylated. Two well-known examples are coagulation factors. Plasma-derived factor IX is fully phosphorylated at serine 158, but recombinant factor IX is not phosphorylated [125], suggesting that ectokinases in CHO cells may be insufficiently expressed. Although the absence of phosphorylation modification does not affect *in vitro* clotting activity, the effect on the *in vivo* clotting activity remains unknown. Factor VIII is also found phosphorylated when exposed to activated platelets [126]. Similar data was also obtained for factor V. Phosphorylation of these two cofactors seems to downregulate their activities, since partially phosphorylated factor Va appears to be more sensitive to activated protein C inactivation [36]. Biologically active peptide hormones, such as adrenocorticotropin [127] and progastrin [128], are found phosphorylated. It would be of interest to know if FAM20C or four-jointed is the right ectokinase responsible for phosphorylating these proteins *in vivo*.

### 3.5. Amidation

Amidation is a replacement of C-terminal carboxyl group of a protein with an amide group. This is a typical modification of many bioactive peptides for full activity [129, 130]. Amidation is ubiquitously found in higher eukaryotes but not in yeast or prokaryotes. Peptide hormones are produced as glycine-extended intermediates, and then the glycine N–C alpha bond is oxidatively cleaved to form active amidated hormones.

Amidation reaction is catalyzed by two enzymes: peptidylglycine *α*-hydroxylating monooxygenase (PHM) and peptidyl-*α*-hydroxyglycine *α*-amidating lyase (PAL). In mammals and higher organisms, these enzymes are expressed as a single bifunctional enzyme, peptidylglycine alpha-amidating monooxygenase (PAM) [130]. The PHM catalyzes the reduction of molecular oxygen for the hydroxylation of the glycine *α*-carbon of glycine-extended substrates, with two redox-active coppers reduced by ascorbate. The PAL produces *α*-amidated peptide product and glyoxylate by cleaving the glycine N–C alpha bond from the peptidyl *α*-hydroxyglycine intermediate. The PAM enzyme localizes in the distal trans-Golgi network through its cytosolic domain [131].

The exact biological roles of *α*-amidation remain to be fully elucidated. This modification prevents ionization of the C-terminus of peptides. It may therefore render it more hydrophobic and better bind to its receptor [130]. It may also contribute to peptide stability. Recombinant salmon calcitonin that regulates serum calcium and phosphate levels is amidated [132]. The product (Forcaltonin) is for the treatment of Paget's disease and hypercalcaemia. It is produced in *E. coli* as a fusion protein and needs downstream *in vitro* modification with a recombinant *α*-amidating enzyme. It is known that CHO, COS-7, and NIH-3T3 can allow amidation of secretory products with an engineered C-terminal glycine [133, 134]. Therefore recombinant bioactive peptides and their fusion proteins could presumably be produced with an *in vivo* approach. 

## 4. N-Linked Glycosylation

N-linked glycosylation is the most common posttranslational modification found in eukaryotic cells, contributing to many crucial biological and physiological roles [111, 135]. The sophisticated modification process involves both compartments of ER and Golgi. The consensus site for N-linked glycosylation is the sequon of Asn-X-Ser/Thr where X can be any amino acid except proline [136, 137]. Acidic amino acids such as aspartate and glutamate can also reduce efficiency. Glycosylation at Asn-Ala-Cys has also been reported [138]. Glycosylation efficiency of these Thr, Ser, and Cys containing sequon is very different with an order of Thr>Ser>Cys [139]. Some nonstandard sequons have been recently identified for N-linked oligosaccharide modification in a small percentage of human recombinant antibodies [140].

N-linked oligosaccharides are added to proteins *en bloc* in the lumen of ER as presynthesized core units of 14 saccharides (Glc_3_Man_9_GlcNAc_2_) in virtually all eukaryotes [137, 141]. This core glycan is the product of a biosynthesis pathway in which monosaccharides are added to a lipid carrier (dolichol pyrophosphate) on both sides of ER membranes by monosaccharyltransferases in the membranes. These enzymes are called ALG (for Altered in glycosylation), identified from studies of yeast mutants. The sugar moiety is translocated from cytosolic side to the luminal side of ER by an ATP-independent bidirectional flippase [142]. The oligosaccharyltransferase (OST) then scans the emerging polypeptide from translocon complex for glycosylation sequon and adds the core glycan unit to the side chain nitrogen of the Asn residue by N-glycosidic bond [141, 143]. Three oligosaccharyltransferase complexes have been identified in mammals; each contains one of two Stt3 proteins, the presumed catalytic units. After the core glycan is added to the growing nascent polypeptide chain, the oligosaccharide portion is modified by a series of glycosidases. Glucosidase I and II, located in the ER, remove all three glucose residues from the core unit to produce a Man_9_GlcNAc_2_ high mannose structure. 

This ER modification process choreographs with protein folding cycle of many glycoproteins, in which glycans serve as sorting signals reflecting the folding status of the protein. The monoglucosylated glycans after the action of glucosidase I and II are bound to two ER lectin chaperones, calnexin or calreticulin, which prevents protein aggregation and ER exit of partially folded proteins [136]. This binding exposes the glycoprotein to the binding of ERp57, the protein disulfide-isomerase A3, for protecting from nonproductive disulfide bonding. When glucosidase II removes the remaining glucose residue, the glycoprotein no longer binds to the lectins and is free to exit ER unless recognized by UDP-Glc:glycoprotein glucosyltransferase (GT). GT can reglucosylate incompletely folded glycoproteins and reinitiate the lectin chaperone binding. The repeated cycles of GT and glucosidase II facilitate efficient folding of newly synthesized glycoprotein. The unsalvageable misfolded proteins are subjected to the ER-associated degradation process [136]. For correctly folded-glycoproteins, many of them are further acted by ER *α*-mannosidase I, before exiting ER, to have terminal mannose removed from the central arm of Man_9_GlcNAc_2_.

In the *cis*-Golgi, *α*-1,2 mannosidase IA, IB, IC further trim 4–6 terminal mannose to produce a Man_5_GlcNAc_2_, a key intermediate for hydrid and complex N-glycans [141]. An N-acetylglucosaminyltransferase named GnTI in *medial*-Golgi adds a GlcNAc residue to C-2 of the mannose *α*1-3 in the core of Man_5_GlcNAc_2_. After this step, majority of N-glycans are trimmed by *α*-mannosidase II to form GlcNAcMan_3_GlcNAc_2_. Then a second GlcNAc is added by the action of GnTII to yield the precursor for all biantennary complex N-glycans. Those glycans not digested by *α*-mannosidase II become precursors of hybrid N-glycans. A “bisecting” GlcNAc can be added to the core *β*-mannose by GnTIII enzyme for both complex and hybrid N-glycans. Further sugar additions and maturation, most of which occur in the *trans*-Golgi, add fucose, galactoses, GlcNAc, sialic acids, and sulfate to convert limited glycan repertoire into an extensive array of mature complex oligosaccharides [111].

N-glycans are most commonly found in therapeutic proteins [9, 10]. The presence and nature of the oligosaccharides clearly affect these proteins' folding, stability, trafficking, immunogenicity, and their primary activities. It is well known that the N-glycans at Asn297 in Fc region of recombinant antibodies and Fc fusion proteins are critical to the activation of downstream effector mechanisms. The Fc oligosaccharides are different from those typical N-glycans and are predominantly fucosylated nongalactosylated diantennary oligosaccharides, with a small portion of galactosylated or sialylated diantennary oligosaccharides. The difference may be due to the inaccessibility of Fc N-glycan for further modification, as the N-glycans at the Fc regions are integral to the IgG structure and have a defined conformation [144]. Besides Fc core glycans, about 30% of polyclonal human IgG molecules contain N-linked oligosaccharides within the IgG-Fab region [144]. Therapeutic antibody cetuximab has an N-linked glycan at Asn88 of the heavy chain variable region and an unoccupied N-linked motif at Asn41 of the light chain variable region [145]. Fab oligosaccharides are more a normal type of N-glycans with heterogeneous complex diantennary and hybrid oligosaccharides with sialic acids and galactoses. 

N-glycans also affect many nonimmunoglycoproteins such as growth factors, cytokines, hormones, and therapeutic enzymes. Removal of either two or all three N-linked sites of human EPO results in poor product secretion [146]. Inhibiting glycosylation decreases biological activity and circulatory half-life of interferon (IFN)-*β* and IFN-*γ*, even though glycosylation is not essential for INFs protein efficacy or safety [147]. Oligosaccharide structures of follicle-stimulating hormone heterodimer play an important role in its biosynthesis, secretion, metabolic fate, and functional potency [148]. The glycans at each subunit seem to exhibit distinct roles, with those in *α* subunit being critical for dimer assembly, signal transduction, and secretion, and those in *β* subunit being more crucial for circulation clearance. In addition, many therapeutics enzymes such as recombinant human glucocerebrosidase for Gaucher disease [68] are glycoproteins and N-glycosylation is important for targeting and functional activities. Factors V, VIII, IX, and X contain N-linked glycosylation [36]. Removing N-glycans from recombinant factor IX drastically increases its specific activity. N-linked glycosylation is essential for the secretion of both factors V and VIII.

## 5. Exocellular Modifications

Soon after therapeutic proteins complete the trafficking pathways and reach cell surface, they are released into extracellular medium environment and incubated there during the production period. Then they are purified, formulated, and stored. They can go into test subjects almost exclusively through parenteral routes such as intravenous, subcutaneous, or intramuscular injection [149]. Systemic circulation allows these proteins to be limitedly distributed into various tissues. Therapeutic proteins are then metabolized and eventually cleared out of the test subjects. A number of modifications occur to proteins when they are in the extracellular media, during *in vitro* manipulation, and *in vivo* circulation. 

### 5.1. Deamidation

Nonenzymatic deamidation of asparagine and glutamine residues to aspartate and glutamate is a common modification for proteins [150, 151]. It is a hydrolytic reaction with water to form products through the formation of succinimide intermediate [151, 152]. Deamidation of Gln is slower than that of Asn. The deamidation rate is affected by conditions of pH, temperature, and ionic strength [151]. The rate also depends on the sequence around Asn and Gln. Polar residues preceding Asn and Gln, neighboring with Ser and Thr, can increase deamidation rates. Asn followed by Gly is found to be the most susceptible site [152]. Secondary, tertiary, and quaternary structures also influence deamidation reaction.

Deamidation of Asn and Gln contributes to charge heterogeneity of therapeutic proteins, since more acidic species are introduced by adding one negative charge and by decreasing isoelectric point [15, 151]. It has been shown that deamidation of Asn and Gln determines the irreversible thermal denaturation of proteins at acidic and neutral pH. Deamidation also affects protein crystallizability and X-ray diffraction due to microheterogeneity. Deamidation regulates the rate of protein breakdown, and could shorten *in vivo* half-life [151]. Both human insulin and growth hormone have been found to deamidate slowly in different formulations, affecting biological potency [151]. Deamidation commonly occurs for recombinant monoclonal antibodies under various conditions and at various domain regions such as CDRs, CH1-3, and CL [15], contributing to heterogeneity and instability, and presumable efficacy.

### 5.2. Glycation

Glycation is a nonenzymatic condensation reaction between a carbonyl group on reducing sugars and N-terminal primary amine or the amine group of lysine side chains [153]. Glycation can occur to physiological proteins in human body where it is implicated in the pathogenesis of multiple chronic diseases [154, 155].

It has been shown that glycation can happen to recombinant antibodies during cell culture [156, 157], which generates product heterogeneity and may affect product stability [158]. Glycation to various antibodies can also take place *in vitro*, such as storage with lactose [159]. Glycation can disguise positive charges of N-terminal primary amino group or side chains of lysine residues, making the antibody more acidic [15]. It can reduce immunoreactivity of antibodies [160]. It has been reported that lowering the concentration of glucose in the fedbatch culture can control the glycation level of a recombinant antibody in CHO cells [78]. 

### 5.3. N-Terminal Pyroglutamate Formation

It has been well known that N-terminal Gln residue can readily cyclize with its own terminal amino group to form pyrrolidone carboxylic acid [161]. It remains uncertain whether or not the conversion reaction is nonenzymatic [162, 163]. Pyroglutamate is frequently detected in heavy chain and light chain of antibodies. N-terminal residues of human IgG heavy chains typically start with either glutamine or glutamate [164], while Lambda light chains begin with glutamine occasionally with serine, arginine, or leucine [165] and Kappa light chains with glutamate or aspartate [166–168]. The N-terminal glutamate can also be cyclized to form pyroglutamate.

The formation of N-terminal pyroglutamate from Gln and Glu decreases molecular mass by 17 and 18 Da. It also causes a loss of positive charge at neutral pH from a loss of the N-terminal primary amine, making the antibody more acidic [15]. Antibody with pyroglutamate elutes earlier than those with glutamine on cation exchange column. Pyroglutamate formation is found resistant to amino peptidases and therefore is proposed as a stabilization mechanism [169]. It has been recently demonstrated that engineering an N-terminal pyroglutamate to a peptide-antibody bispecific genetic fusion molecule can restore its IL17A neutralizing activity in a cell-based assay by preventing N-terminal degradation [53].

### 5.4. Oxidation

Methionine, one of the most susceptible residues for oxidation, can be oxidized to methionine sulfoxide for therapeutic proteins such as antibodies [15] and growth factors [170]. This increases the mass by 16 Da and makes the side chain of Met more polar. Met255 and Met431 in the Fc region of human IgG_1_ are two most susceptible sites for oxidation when incubated at elevated temperatures [171]. These two residues are close to the CH2-CH3 interface [172], making them more susceptible. Antibodies with oxidized Met elute earlier relative to nonoxidized molecule on HIC column [171].

In addition to Met residues, Trp and Cys amino acids are also susceptible to oxidation. Oxidized Trp residues have been reported to be located in the CDRs regions for several antibodies [173–175]. Cys residues of antibodies are typically involved in disulfide bonding, but oxidation of unpaired Cys of a murine antibody is reported after a long storage [176]. Oxidation of histidine and tyrosine is also reported [177].

### 5.5. Proteolytic Processing

Proteolytic processing is a common modification in the extracellular space. Besides the nonspecific protein degradation mediated by various plasma and tissue proteases, some proteolytic processings are explicitly required for enzyme activation. Native protein of recombinant activated protein C, Xigris, is produced and circulated as a zymogen which is activated by thrombin in complex with thrombomodulin [178]. In plasma, factor VIII is activated by thrombin by the cleavage initially after Arg740 and then after Arg372 and Arg1689 to produce a heterotrimer [36]. It can be inactivated by activated protein C at Arg336 and Arg562. Factor V is activated by Thrombin at Arg739, Arg1018, and Arg1545, and inactivated by activated protein C at Arg306 and Arg506 [36]. Factor IX is activated by factor XIa to remove activation peptide. Factor X is activated by the cleavage of the tissue factor/factor VIIa complex or the factor VIIIa and factor IXa complex.

C-terminal lysine removal of antibody heavy chain is a common modification of recombinant antibodies. It decreases the molecular mass by 128 Da and 1 unit of positive charge. Mammalian cell lines such as CHO, NSO, and SP2/0, produce an endogenous carboxypeptidase B that can result in this processing [179]. Partial removal of this residue can cause heterogeneity.

## 6. Biopharmaceutical Applications of Protein Modifications 

Posttranslational modifications have been utilized as strategies for therapeutic improvements [9, 10, 144]. A better understanding of structure and function relationship as well as molecular pathways has facilitated the development of next generation of biotherapeutic products. The following summarizes the recent advances in protein engineering, cell line engineering, and process engineering, for improving functional characteristics and attributes of biopharmaceutical products (Table 2).

### 6.1. Applications in Protein Engineering

Engineering disulfide bonds by introducing cysteines into proteins have long been a protein engineering strategy for stabilizing small proteins and multiunit proteins [180–183]. In order to prolong the activity of factor VIIIa, Tyr664 and Thr1826 are mutated to Cys to introduce a disulfide bond between A2 and A3 domains [46]. The resulting protein significantly prolongs factor VIIIa stability and activity. A similar result is obtained for factor Va when a disulfide bond between its A2 and A3 domain is introduced [184]. Disulfide bond has also been introduced to framework region of antibody Fv fragments to improve stability [47]. It improves stability of IgG-like bispecific scFv antibody [185]. Also an *in vivo* cleavable disulfide linker has been designed for *in vivo* release of granulocyte colony-stimulating factor from its transferrin fusion [186].

Interchain disulfide bonds of IgG_1_ can be distinguished by chemical reduction and oxidation method [187]. Cytotoxic drugs are therefore conjugated to antibodies through these activated cysteine sulfhydryl groups [188]. To resolve the issue of heterogeneity and to improve therapeutic index (defined as the lethal dose of a drug for 50% of the population divided by the minimum effective dose for 50% of the population), cysteine substitutions at positions of light chain and heavy chain have been applied successfully for site-specific conjugation [189, 190].

One of the best known examples making use of protein modification, the so-called glycoengineering, is the half-life extension of Aranesp [48, 191]. After introducing 48 N-linked positions and evaluating 62 constructs, the terminal half-life of Aranesp with two additional N-glycans is approximately 3-fold longer than that of wild-type Epoetin Alfa when administered intravenously. This is due to the fact that glycosylation can shield nonspecific proteolytic degradation, reduce renal clearance by negatively charged sialic acids at glycan chain termini, and decrease kidney glomerular filtration by increased molecular weight and hydrodynamic radius of the glycoprotein.

Glycoengineering has also been applied to improve the solubility and physicochemical properties of therapeutic proteins such as antibodies. Introducing N-linked glycans into antibody framework regions can increase protein solubility by more than 50-fold [49]. Similar results are obtained when an N-linked site is introduced to the CDR region [50].

O-linked glycan has also been applied to protein engineering. The carboxyl-terminal peptide (CTP) of human chorionic gonadotropin-*β*-subunit [52] contains several proline and serine residues and four O-linked oligosaccharides sites. When this CTP sequence is fused to human follitropin [192], erythropoietin [51], or human growth hormone [193], it increases the half-life of the fusion proteins in the circulation. This is also presumably due to the negatively charged sialic acids on the O-linked glycans that decrease renal clearance and glomerular filtration.

Modifying proteolytic processing is another strategy for improving therapeutic efficacy. Engineering an N-terminal glutamine to form pyroglutamate has been successfully applied to restore cellular activity of a peptide-antibody fusion by preventing N-terminal protein degradation [53]. Modifying proteolytic specificity of activated protein C toward serine protease inhibitor family proteins has helped improve their pharmacokinetic profiles [194]. Pipe and Kaufman generated an protease resistant FVIIIa by eliminating two APC cleavage sites at Arg336 and Arg562 [54]. 

### 6.2. Applications in Cell Line Engineering

Genetic engineering of host cell lines has proven to be a practical tool to obtain therapeutic proteins with improved properties. Due to the importance and prevalence of glycosylation on therapeutic proteins, a significant progress in cell line engineering by utilizing glycosylation pathways has been made for the past two decades [9, 10, 144, 195, 196]. 

Terminal capping of glycans with sialic acids improves *in vivo* circulation half-life of glycoproteins because of negative charge and unrecognization of asialoglycoproteins and mannose receptors. A number of cell line engineering efforts have been put in place. Overexpressing GnTIV and GnTV have been found to increase glycan proportion with triantennary and tetra-antennary structures [197, 198]. Further overexpressing ST3 *β*-galactoside *α*-2,6-sialyltransferase IV (ST3GalIV) and/or ST6 *β*-galactoside *α*-2,6-sialyltransferase I (ST6GalI) increases sialylation up to 80%. ST6GalI, which transfers sialic acid in an *α*2,6-linkage, is not expressed in CHO. Overexpressing this gene increases the *α*-2,6-linked sialic acid over *α*-2,3-linked sialic acid, which is reported to improve circulation half-life [199, 200]. Overexpression of both *β*-1,4-galactosyltransferase and ST3Gal also improves sialylation [58, 201].

Sialidase can degrade sialic acid when released into the culture due to cell lysis [202, 203]. Sialidase knockdown with stable antisense or siRNA in CHO is found to increase sialic acid content of recombinant glycoproteins [59, 60]. Overexpressing CMP-sialic acid transporter is shown to improve IFN-*γ* sialylation [56]. Overexpressing a key enzyme GNE for sialic acid synthesis can also improve sialylation of recombinant EPO [57]. A combinatorial approach with ST3Gal, CMP-sialic acid synthase, and CMP-sialic acid transporter, is found to better improve sialylation than overexpressing individual gene alone [55, 58].

On the other end, to generate antibody with reduced sialylation, the catalytic domain of the arthrobacter ureafaciens sialidase is stably expressed in mammalian cells [70]. The soluble sialidase A is secreted into medium where it is capable of removing sialic acid thoroughly from antibodies. The low sialylated antibody possesses improved antibody-dependent cellular cytotoxicity (ADCC) activity.

N-acetylneuraminic acid (Neu5Ac) can be converted into N-glycolylneuraminic acid (NeuGc) by CMP-sialic acid hydroxylase. NeuGc is not produced by human and therefore immunogenic, antisense knockdown of CMP-sialic acid hydroxylase has been engineered in CHO to reduce NeuGc down to 1% [61].

O-linked glycan such as sialyl Lewis X (sLe^x^) in E-Selectin protein is important for mediating cell adhesion. This glycan formation can be promoted by overexpressing *β*1,6-N-acetyl-glucosaminyl-transferase (C2GnT) and fucosyltransferase 6 (Fut6), as well as downregulating ST3Gal [62, 63].

Another well-known direction of cell line engineering in glycosylation is modulating antibody effector function [144, 204]. N-glycans in the Fc-region play a critical role in ADCC activity. Absence of a core *α*-1, 6-linked fucose improves binding to Fc*γ*RIII and the *in vitro* ADCC activity [205, 206]. Defucosylated anti-CCR4 antibody mogamulizumab (KW0761) has been approved in Japan for relapsed adult T-cell leukemia-lymphoma [123]. Knockingdown *α*-1,6-fucosyltransferase (Fut8) [65] and GDP-mannose 4,6-dehydratase (GMD) with siRNAs [64], homologous recombinant fut8 knockout [67], zinc-finger nucleases fut8 knockout [207], or overexpressing N-acetyl-glucosaminyltransferase III (GnTIII) [66] have been utilized for modifying fucosylation of IgG Fc glycans. Glycoengineered anti-CD20 antibody obinutuzumab (GA101) is currently used in clinical studies [208]. Inactivating GnTI in CHO cells (*Lec1*) also generates a fucose-free oligomannose glycan with enhanced ADCC and decreased complement-dependent cytotoxicity (CDC) activities for IgG [69]. Oligomannose glycan generated by the *Lec1* mutant has been employed for targeted delivery to disease-affected tissues for the treatment of lysosomal storage diseases [68].

Besides glycosylation, cell line engineering has been applied for improving carboxylation modification. In order to improve carboxylation rate of factor X, a HEK293 line with overexpressing vitamin K epoxide reductase is constructed [71]. It has been shown that factor X in such a cell line is 92% carboxylated, up from control 52%. Interestingly, overexpressing the GGCX, the carboxylase, has no improvement for the modification. It is presumably due to the fact that the reduction of vitamin K epoxide to vitamin K and the conversion of vitamin K to vitamin K hydroquinone are the rate-limiting steps under the culture condition. Similarly, overexpressing VKOR in HEK293 cells together with shRNA knockdown of calumenin (an inhibitor of VKOR and *γ*-carboxylase) increases the fraction of bioactive factor VII by sevenfold [72]. Recombinant factor IX is produced by a CHO line engineered to coexpress Furin protein [209]. This modification enhances propeptide removal and consequentially the factor IX activity, as failure to remove propeptide prevents the Gla domain from adopting proper calcium-induced conformation.

### 6.3. Applications in Process Engineering

A number of bioprocessing parameters can influence protein modification such as glycosylation [210]. They include serum content, culture pH, dissolved oxygen, temperature, growth phase, ammonium concentration, and salt concentration [75, 210–218]. Glycan degradation mediated by exoglycosidases such as sialidase and galactosidase also affects product consistency through cell viability and growth condition [202, 219]. Site occupancy for N- and O-linked glycosylation varies according to culture condition. These effects on modification have been attributed to availability of dolichol phosphate and nucleotide sugars [220], glycotransferase activity [221, 222], and potential modification site competing with protein folding [223, 224]. Ammonium is a common metabolic waste product driven by a major energy source of glutamine or asparagines. Increased ammonia levels in the cell culture can increase the pH of trans-Golgi compartment, therefore compromise pH-sensitive glycotransferases activity [215, 225].

Several strategies in process engineering have been developed to improve product quality and attributes. Glucosamine and uridine supplementation into CHO cell culture has led to an elevated level of intracellular UDP-GlcNAc and enhanced glycan antennarity [73]. Addition of CMP-Neu5Ac precursor, N-acetylmannosamine (ManNAc), can improve sialylation of IFN-gamma [76]. Feeding specific lipid supplements to cultures has been found to reduce the deterioration in site occupancy of IFN-gamma during batch culture [74]. OST enzyme requires manganese for maximal activity, therefore supplement with additional manganese or iron can increase glycan site occupancy of human tissue plasminogen activator [75]. Manganese addition to the production process of human EPO can also increase galactosylation and reduce lower sialylated fraction [226]. Decreasing culture temperature from 37°C to 33°C or 31°C, and adding butyrate or thyroid hormones, can increase glycan occupancy rate. Both butyrate addition and lowering temperatures decrease cell growth and therefore slow down the protein elongation rate which increases the exposure time of glycan site to OST enzyme in ER. Trace amount of metals affects glycosylation. Addition of copper (II) chloride is found to increase sialic acid content [218, 227]. To generate nonfucosylated oligomannose modified antibody, Zhou et al. added a potent *α*-mannosidase I inhibitor, kifunensine, to CHO cells for 11 days in batch culture [77]. The resulting antibody shows a greater affinity to Fc*γ*RIIIA, a higher ADCC activity, a lower binding to C1q, and a lower CDC activity. Glycation is a source of heterogeneity for recombinant therapeutic proteins. Yuk and coworkers demonstrate that by lowering the concentration of glucose from 6 g/L down to 3 g/L, >40% reduction in glycation is observed [78]. A >80% reduction is further generated with a fully continuous glucose fed at ~1 g/L.

## 7. Conclusions and Perspectives

The importance of protein modifications and their underlying mechanisms has become a core and focus of both industrial and basic research investigations. They can potentially affect protein production level, stability, pharmacokinetics/pharmacodynamics parameters, and immunogenicity. The functional and safety consequences of any of these modifications to a therapeutic protein must be evaluated on an individual protein basis. Advances in understanding the relationship between modification structure and function have enabled the rational designs to enhance specific functional features as demonstrated by the studies previously mentioned. Also this knowledge contributes to imposing strategic bioprocessing controls to ensure consistency of product quality. While glycosylation represents the most widespread and complex modification, other protein modifications such as phosphorylation and sulfation attract increasing attentions in recent years. Knowledge gaps remain for the understanding of the biology of these modifications. A recent report on the draft genome sequence of an ancestral CHO line [228] opens a new door for feasibly manipulating gene products involved in protein modification pathways. A further fundamental understanding of therapeutic protein modifications will culminate in the development of many new generations of biopharmaceutical products with preselected modifications and enhanced properties.

## Figures and Tables

**Table 1:** Consensus motifs and enzymes responsible for therapeutic modifications.

Modification type	Linkage	Consensus sequence	Enzymes
N-glycosylation	GlcNAc-Asn	N-X-(S/T), X≠Pro	OST
Mucin-type O-glycosylation	GalNAc-Ser/Thr	No consensus, Pro favorable	ppGalNAcT
Disulfide bond	–S–S–	Cysteine pairs	Ero1-PDI
*γ*-carboxylation	Glu→Gla	Mediated by adjacent propeptide	GGCX-VKOR
*β*-hydroxylation	Asn→Hyn; Asp→Hya	C-X-D/N-X-X-X-X-F/Y-X-C-X-C but not sufficient	*β*-hydroxylase
Tyrosine sulfation	O4-sulfate ester	No simple consensus site Glu/Asp around Tyr favorable	TPST1/TPST2
Propeptide cleavage	R/K ↓	With single or pair of basic amino acids	PC family members
Phosphorylation	pS, pT	S-x-E/pS or Ser/Thr	FAM20C or four-jointed
Amidation	C-terminal carboxyl→amide	C-terminal glycine	PAM
Deamidation	Asn→Asp; Gln→Glu	Asn-Gly most susceptible	Unknown or none
Glycation	Ketoamine or Amadori product	N-terminal primary amine Or amino group of lysine side chain	Unknown or none
Pyroglutamate	Pyrrolidone carboxylic acid	N-terminal Gln or Glu	Unknown or none
Oxidation	Met→Met sulfoxide	Met (Trp, Cys, Tyr, His)	Unknown or none
Proteolytic processing	Arg ↓ or ↓ Lys	Basic amino acid	APC, carboxypeptidase B

**Table 2:** Biopharmaceutical applications of therapeutic protein modifications.

Applications	Function/gene	Mechanism of action	Reference
(1) Protein engineering			

Disulfide bond introduction	Cysteine engineering	Stabilizing FVIIIa	[46]
		Stabilizing scFV	[47]
N-linked site introduction	Half-life extension of EPO	Reducing renal clearance	[48]
	IgG framework/CDR	Improving solubility	[49, 50]
Poly O-linked sites	Fusion of CTP of gonadotropin	half-life extension	[51, 52]
Engineering N-terminal Gln	Forming pyroglutamate	Preventing N-terminal degradation	[53]
Protease resistant mutant	Mutating Arg336 & 562 of FVIII	Eliminating two APC sites	[54]

(2) Cell-line engineering			

	Overexpressing GnTIV, GnTV, ST3GalIV, and ST6GalI	Increasing sialic acid content	See text
Half-life extension	CMP-sialic acid transporter		[55, 56]
	CMP-sialic acid synthase, GNE		[57, 58]
	Knockdown sialidase	Preventing sialic acid degradation	[59, 60]
Reducing immunogenicity	knockdown CMP-sialic acid hydroxylase	Preventing conversion of Neu5Ac to Neu5Gc	[61]
E-selectin interaction	Overexpressing C2GnT, Fut6 Knockdown ST3Gal	Enhancing sLe^x^ formation	[62, 63]
Modulating effector function	Knockout or knockdown	Fucose removal	[64, 65]
	Fut8, GMD, overexpressing GnTIII		[66, 67]
Lysosomal targeting	Inactivating GnTI	Oligomannose formation	[68, 69]
Improving ADCC	Overexpressing sialidase	Reducing sialylation	[70]
Improving carboxylation	Overexpressing VKOR	Facilitating vitamin K reduction	[71]
	VKOR ↑ calumenin ↓	Increasing carboxylation of FVII	[72]

(3) Process engineering			

Modulating glycosylation	Serum content, pH, dissolved oxygen, Temperatures, ammonium, salt concentration	Bioprocessing parameters	See text
Glucosamine/uridine	Enhancing glycan antennarity	Elevating intracellular UDP-GlcNAc	[73]
Lipid supplement	Maintaining site occupancy		[74]
Decreasing temperature/adding butyrate	Enhancing glycan occupancy	Increasing site exposure time to OST	[75]
Addition of ManNAc	Improving sialylation	CMP-Neu5Ac precursor	[76]
Adding manganese/iron	Enhancing glycan occupancy	Increasing OST activity	[75]
Addition of kifunensine	Oligomannose formation	*α*-mannosidase I inhibitor	[77]
Lowering glucose	Reducing heterogeneity	Reducing glycation	[78]
